# *Urtica dioica* L. Leaf Extract Dose-Dependently Modulates Oxidative Stress in the Kidney and Exerts Anti-Fibrotic and Anti-Inflammatory Properties by the Molecular Mechanisms Independent of NRF-2 Signalization Mirroring the Effects of Losartan in SHR

**DOI:** 10.3390/ijms252413272

**Published:** 2024-12-11

**Authors:** Una-Jovana Vajic, Nevena Mihailovic-Stanojevic, Danijela Karanovic, Maja Zivotic, Milan Ivanov, Djurdjica Jovovic, Jelica Grujic-Milanovic, Zoran Miloradovic

**Affiliations:** 1 Department for Cardiovascular Physiology, Institute for Medical Research, National Institute of Republic of Serbia, University of Belgrade, Dr Subotića 4, P.O. Box 39, 11129 Belgrade, Serbia; unajovana@imi.bg.ac.rs (U.-J.V.); danijela.karanovic@imi.bg.ac.rs (D.K.); ivmilan@imi.bg.ac.rs (M.I.); djurdjica@imi.bg.ac.rs (D.J.); jeca@imi.bg.ac.rs (J.G.-M.); zokim@imi.bg.ac.rs (Z.M.); 2 Institute of Pathology, Faculty of Medicine, University of Belgrade, Dr Subotića 1, 11000 Belgrade, Serbia; majajoker@gmail.com

**Keywords:** *Urtica dioica* L., hypertension, oxidative stress, kidney, nitric oxide, collagen, fibronectin, plasminogen activator inhibitor 1, nitro-tryptophan, Nrf-2 signaling

## Abstract

Previously, we confirmed systemic antihypertensive and antioxidant properties of *Urtica dioica* L. leaf extract (UE) in spontaneously hypertensive rats (SHR). Here, we aimed to evaluate whether UE can alter the NO and Nrf-2 signaling to prevent local oxidative stress and kidney damage in the model of essential hypertension. SHR were divided into five groups: SHRC-control, received 0.5 mL/day of water, SHR+L received 10 mg/kg/day of losartan, SHR+UE10, SHR+UE50, and SHR+UE200 received 10, 50, and 200 mg/kg/day during next 4 weeks. At the end of the experiment, urine samples were collected for albuminuria and nitrate/nitrite assessment. Mean arterial pressure (MAP) was measured, and blood samples were collected for plasma creatinine evaluation. Kidneys were analyzed for nitrate/nitrite, oxidative stress, and target molecules by biochemical, Western blot, and immunofluorescent techniques. Losartan and UE50 significantly reduced MAP, albuminuria, oxidative stress, fibroinflammatory markers, and NRF-2/CAT/SOD signaling, with a significant increase in 6-nitrotryptophan and eNOS expressions compared to control. The effects of UE showed dose dependence. Beneficial effects of UE and losartan were independent of NRF-2 signalization in SHR. Interestingly, all treatments induced the increase in 6-nitrotryptophan expression, thus further studies are needed to elucidate the mechanisms of such nitrated tryptophan.

## 1. Introduction

Systemic hypertension and cardiovascular pathogenesis are considered chronic and slowly evolving conditions that progress throughout life, often culminating in heart failure, stroke [1], and most importantly, progressive kidney disease [2].

Nitric oxide (NO) plays a key role in the blood pressure regulation [3]. Impaired NO bioavailability is an important feature of hypertension [4]. In addition, oxidative stress, an excessive generation of reactive oxygen species (ROS), is present in hypertension and overproduced ROS are known as NO scavengers that lead to its reduced overall bioavailability [4]. NO is formed from L-arginine by the activity of different isoforms of the NO synthase, the endothelial (eNOS), neuronal (nNOS), and inducible NO synthase (iNOS) [5]. The second source of NO is organic nitrates that have long been used in clinical practice to induce vasodilation, as nitrates and nitrites are the main substrates for NO production via the NOS-independent pathway [3], but nitrate tolerance and increased formation of peroxynitrites under oxidative stress limit their use. Nuclear factor erythroid factor 2-related factor 2 (Nrf2) is a transcriptional regulator of cellular defense against oxidative stress that has been shown to ameliorate renal damage by eliminating ROS [6]. It does not have antioxidative function per se but exerts antioxidant effects by activating the transcription of target antioxidant genes including the enzymes superoxide dismutase and catalase [7].

Hypertension is associated with an increase in the deposition of extracellular matrix (ECM) (especially collagen types I, III, and IV) in the renal resistance vessels, glomeruli, and interstitium [8]. Therefore, renal fibrosis is another important complication associated with the progression of hypertension, where the abnormal accumulation of collagens contributes to vascular remodeling and the progression of renal damage [9]. Type IV collagen is commonly used as a marker of glomerular sclerosis and interstitial fibrosis [10], and in spontaneously hypertensive rats (SHR), remodeling of the ECM is associated with these fibrogenic responses [8]. Although many cell types in the kidney can produce ECM, (myo)fibroblasts in the interstitium and mesangial cells in the glomeruli are thought to be the main cellular mediators of interstitial fibrosis and glomerulosclerosis [10]. Myofibroblasts, cells expressing α-smooth muscle actin (α-SMA), share features with fibroblasts and smooth muscle cells, and are involved in wound contraction and healing [11]. Positive immunostaining for α-SMA has been demonstrated in the glomeruli and interstitium in experimental animal models and in humans with progressive renal insufficiency [11]. Fibronectin, the first ECM protein to be deposited during fibrogenesis, activates integrins, acts as a fibroblast chemoattractant, and colocalizes collagen formation [10]. Another molecule that causes an increase in the ECM accumulation is plasminogen activator inhibitor 1 (PAI-1) [12]. It is known that angiotensin II, the main effector peptide of the renin-angiotensin system, can have a profibrotic effect after binding to the angiotensin II type 1 (AT1) receptor in hypertension, directly or via the induction of transforming growth factor-beta 1 (TGF-ß1) and/or PAI-1; consequently, AT1 receptor blockade reduces renal PAI-1 expression induced by angiotensin II infusion [12,13].

Previous studies have shown that different *Urtica dioica* L. leaf extracts (UE, e.g., prepared from aerial parts or roots) exhibit vasodilatory effects in vitro [14,15] and hypotensive and antihypertensive effects in vivo [15,16,17,18]. Vajic et al. showed that UE is rich in chlorogenic acid, 2-O-caffeoyl malic acid, and rutin, and that these phenolic compounds and their metabolites are responsible for antihypertensive and systemic antioxidant response to UE treatment by the direct antioxidant activity [19,20]. Additionally, we showed that UE is a potent metal ion chelator compared to other plant extracts and that it could potentially prevent oxidative damage caused by Fenton’s reaction [20]. Furthermore, another study from our group pointed out strong DPPH scavenging activity of UE ranging from 0.338 mg/mL to 0.664 mg/mL [21] and it was unaffected by extraction time (30–90 min) or methanol percentage in the solvent (50% *v*/*v* aqueous-methanol). To explain the beneficial effects of *Urtica dioica* L., various mechanisms have been proposed [16,18], such as NO-dependent vasorelaxation [15,18]. However, there are few data on the chronic effects of UE on renal function and structure [22,23].

Since hypertensive nephrosclerosis is a progressive kidney damage caused by long-standing, poorly controlled high blood pressure [24], and taking into account our previous results showing beneficial effects of chronic intake of UE on systolic and diastolic blood pressure as well as systemic oxidative stress by improving systemic antioxidant defense and antioxidant capacity in SHR [19], here we aimed to determine whether supplementation with UE can alter the NO and Nrf-2 signaling and prevent local oxidative stress and the deterioration of kidney function and structure in an experimental model of essential hypertension.

## 2. Results

### 2.1. Body and Kidney Weight, Urine Flow, Blood Pressure, and Kidney Function Parameters

In the SHRL group, MAP was significantly reduced compared to the SHRC group (SHRL vs. SHRC, *p* < 0.01; Table 1). Similarly, higher doses of UE decreased MAP significantly (SHR+UE50 and SHR+UE200 vs. SHRC, *p* < 0.05), while the UE dose of 10 mg/kg/day did not change this parameter (Table 1). Treatment with losartan did not influence BW, KW, Uf, and Albexc (Table 1). Similarly, dietary supplementation of SHR with 10, 50, and 200 mg/kg/day of UE did not affect BW, KW, Uf, and pCr (Table 1). On the other hand, microalbuminuria, measured by urinary Albexc, was significantly reduced in all UE-supplemented groups (SHR+UE10, SHR+UE50, and SHR+UE200 vs. SHRC, *p* < 0.001, *p* < 0.05, and *p* < 0.01, respectively; Table 1).

### 2.2. NO_2_ Content in UE

The detected nitrite concentration in UE was 14.72 mg/g.

### 2.3. Plasma, Urine, and Kidney NO Metabolites

**Figure 1:**
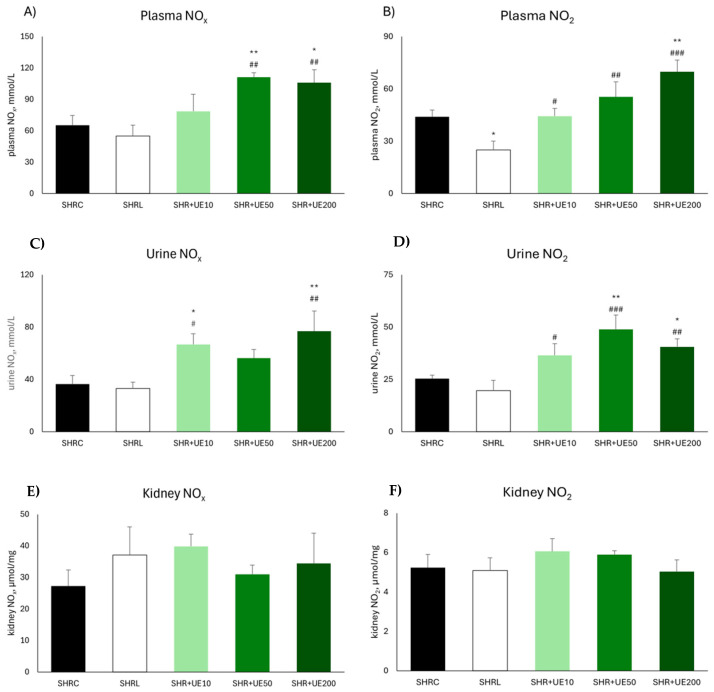
Total (**A**) nitrate/nitrite (NO_x_) and (**B**) nitrite (NO_2_) concentrations in plasma of SHR; total (**C**) nitrate/nitrite (NO_x_) and (**D**) nitrite (NO_2_) concentrations in urine of SHR; total (**E**) nitrate/nitrite (NO_x_); and (**F**) nitrite (NO_2_) concentrations in the kidney of SHR. SHRC received 0.5 mL/day of water, SHR+L received 10 mg/kg/day of losartan, SHR+UE10, SHR+UE50, and SHR+UE200 received 10, 50, and 200 mg/kg/day of *Urtica dioica* L. leaf extract (UE), respectively. * *p* < 0.05 and ** *p* < 0.01 compared to SHRC, # *p* < 0.05, ## *p* < 0.01, and ### *p* < 0.001 compared to SHRL. Data presented as mean ± SEM (one-way ANOVA omnibus test with Fisher least significant difference (LSD) post hoc test, Statistica version 8.0., StatSoft Inc., Tulsa, OK, USA).

Supplementation with 50 and 200 mg/kg/day of UE resulted in a significant increase in plasma NO_x_ value (SHR+UE50 and SHR+UE200 vs. SHRC, *p* < 0.01 and *p* < 0.05, respectively; Figure 1A). Compared to SHRC group, plasma NO_2_ was significantly increased only in the group supplemented with 200 mg/kg/day of UE (*p* < 0.01; Figure 1B). Similarly, urine NO_x_ was significantly increased after supplementation with the lowest and highest UE-supplemented dose (SHR+UE10 and SHR+UE200 vs. SHRC, *p* < 0.05 and *p* < 0.01, respectively; Figure 1C). Likewise, urine NO_2_ was significantly increased after supplementation with 50 and 200 mg/kg/day of UE (SHR+UE50 and SHR+UE200 vs. SHRC, *p* < 0.01 and *p* < 0.05, respectively; Figure 1D). All UE treatments significantly increase urine NO_2_ levels compared to SHRL group (SHR+UE10, SHRUE50, and SHR+UE200 vs. SHRL, *p* < 0.05, *p* < 0.001, and *p* < 0.01, respectively; Figure 1D). Neither of the treatments influenced total kidney NO_x_ nor NO_2_ content (Figure 1E,F). Additionally, in the SHRL group, plasma and urine NO_x_ remained unchanged compared to the values observed in the SHRC group, while NO_2_ concentration was significantly reduced after losartan treatment (SHRL vs. SHRC, *p* < 0.05) (Figure 1B).

### 2.4. The Effects of UE on the Expression of NOS Enzymes and 6-NO_2_Trp in the Kidney of SHR

**Figure 2:**
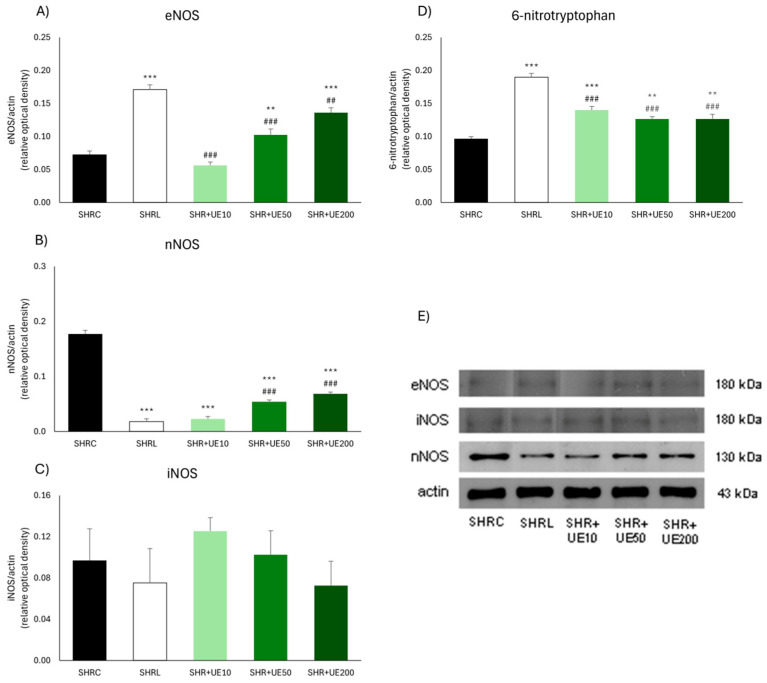
Kidney (**A**) eNOS, (**B**) nNOS, (**C**) iNOS, and (**D**) 6-NO_2_Trp protein expressions in SHR with (**E**) the representative Western blots of NOS isoforms. SHRC received 0.5 mL/day of water, SHR+L received 10 mg/kg/day of losartan, SHR+UE10, SHR+UE50, and SHR+UE200 received 10, 50, and 200 mg/kg/day of *Urtica dioica* L. leaf extract (UE), respectively, ** *p* < 0.01 and *** *p* < 0.001 compared to SHRC, ## *p* < 0.01 and ### *p* < 0.001 compared to SHRL. Data presented as mean ± SEM from three independent experiments (one-way ANOVA omnibus test with Fisher least significant difference (LSD) post hoc test, Statistica version 8.0., StatSoft Inc., Tulsa, OK, USA).

**Figure 3:**
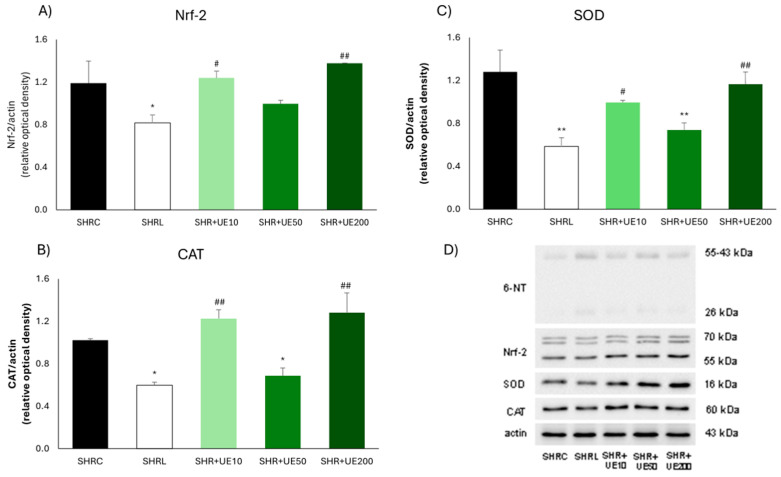
Kidney (**A**) Nrf-2, (**B**) CAT, and (**C**) SOD expressions in SHR with (**D**) representative Western blots. SHRC received 0.5 mL/day of water, SHR+L received 10 mg/kg/day of losartan, SHR+UE10, SHR+UE50, and SHR+UE200 received 10, 50, and 200 mg/kg/day of *Urtica dioica* L. leaf extract (UE), respectively. * *p* < 0.05 and ** *p* < 0.01 compared to SHRC, # *p* < 0.05 and ## *p* < 0.01 compared to SHRL. Data presented as mean ± SEM from three independent experiments (one-way ANOVA omnibus test with Fisher least significant difference (LSD) post hoc test, Statistica version 8.0., StatSoft Inc., Tulsa, OK, USA).

The most pronounced increase in kidney eNOS expression (Figure 2A,E) was observed in the losartan group (SHRL vs. SHRC, *p* < 0.001) and it was also significantly higher than in the 10, 50, and 200 mg/kg UE groups (SHR+UE10, SHR+UE50 and SHR+UE200 vs. SHRC, *p* < 0.001, *p* < 0.001, and *p* < 0.01, respectively). In addition, among the UE-treated groups, there was a trend of a dose-dependent increase in this enzyme. Conversely, compared to the control group, nNOS enzyme expression was significantly decreased in all UE-treated groups (*p* < 0.001; Figure 2B,E), as well as in the losartan-treated group (*p* < 0.001; Figure 2B,E). Additionally, in the case of nNOS expression, a certain dependence appeared among the UE-treated groups, where the expression of this enzyme tended to increase with increasing UE dose (Figure 2B,E). Treatment with two higher UE doses led to significantly higher nNOS expression than in the SHRL group (Figure 2B,E, *p* < 0.001). Regarding iNOS expressions, no difference was observed between the examined groups (Figure 2C,E). The relative abundance of 6-NO_2_Trp (Figure 2D and Figure 3D) increased significantly in all treated groups (SHRL, SHR+ UE10, SHR+UE50, and SHR+UE200 vs. SHRC, *p* < 0.001, *p* < 0.001, *p* < 0.01, and *p* < 0.01, respectively), although this increase was significantly lower after all UE treatments compared to losartan treatment (*p* < 0.001; Figure 2D).

### 2.5. The Effects of UE on the Nrf-2 Signaling Pathway in the Kidney of SHR

UE supplementation did not influence Nrf-2 expression in the kidney of SHR (Figure 3A), while losartan treatment significantly decreased this value compared to SHRC (*p* < 0.05; Figure 3A), as well as compared to SHR+UE10 and SHR+UE200 (SHR+UE10 and SHR+UE200 vs. SHRL, *p* < 0.05; Figure 3A). Compared to the control group, the expression of the CAT enzyme was significantly decreased in the losartan-treated and 50 mg/kg/day UE-supplemented group (*p* < 0.05; Figure 3B), while it remained unchanged in 10 and 200 mg/kg/day UE-supplemented groups (Figure 3B). The level of kidney CAT expression in the losartan-treated group was significantly lower than these levels in the 10 and 200 mg/kg/day UE-supplemented groups (*p* < 0.01; Figure 3B). Similarly, SOD kidney expression was significantly decreased in the losartan-treated and 50 mg/kg/day UE-supplemented group (*p* < 0.01; Figure 3C), while it remained unchanged in 10 and 200 mg/kg/day UE-supplemented groups (Figure 3C). Additionally, the level of SOD expression in the kidney in the losartan-treated group was significantly lower compared to SOD levels in 10 and 200 mg/kg/day UE groups (SHR+UE10 and SHR+UE200 vs. SHRL, *p* < 0.05 and *p* < 0.01, respectively; Figure 3C).

### 2.6. The Effects of UE on Antioxidant Capacity, Lipid Peroxidation, and Protein Oxidation in the Kidney of SHR

**Figure 4:**
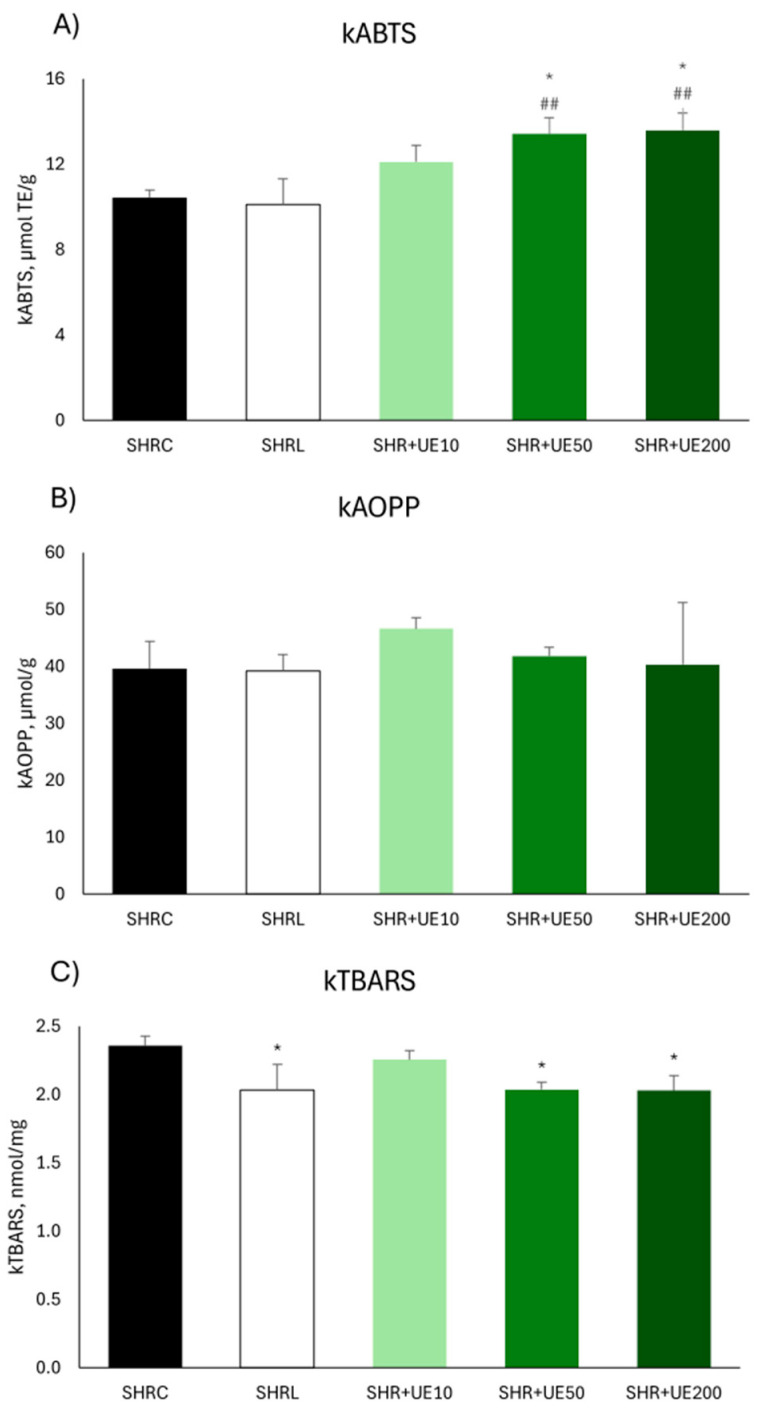
(**A**) antioxidant capacity (kABTS), (**B**) advanced oxidation protein products (kAOPP), and (**C**) lipid peroxidation level (kTBARS) in the kidney of SHR. SHRC received 0.5 mL/day of water, SHR+L received 10 mg/kg/day of losartan, SHR+UE10, SHR+UE50, and SHR+UE200 received 10, 50, and 200 mg/kg/day of *Urtica dioica* L. leaf extract (UE), respectively. * *p* < 0.05 compared to SHRC, ## *p* < 0.01 compared to SHRL. Data presented as mean ± SEM (One-way ANOVA omnibus test with Fisher least significant difference (LSD) post hoc test, Statistica version 8.0., StatSoft Inc., Tulsa, OK, USA).

Antioxidant capacity in the kidney, measured by kABTS, was significantly increased in groups where SHR was supplemented with two higher doses of UE (SHR+UE50 and SHR+UE200 vs. SHRC, *p* < 0.05; Figure 4A), as well as compared to losartan treatment (SHR+UE50 and SHR+UE200 vs. SHRL, *p* < 0.01; Figure 4A). Neither losartan treatment nor UE supplementation influenced protein oxidation in the kidney of SHR (Figure 4B). Supplementation with 50 and 200 mg/kg/day of UE significantly decreased kTBARS level compared to control (SHR+UE50 and SHR+UE200 vs. SHRC, *p* < 0.01; Figure 4C). Losartan treatment also decreased significantly kTBARS compared to control (*p* < 0.01; Figure 4C).

### 2.7. Effects of UE on Kidney Collagen IV, Fibronectin, α-SMA, and PAI-1

**Figure 5:**
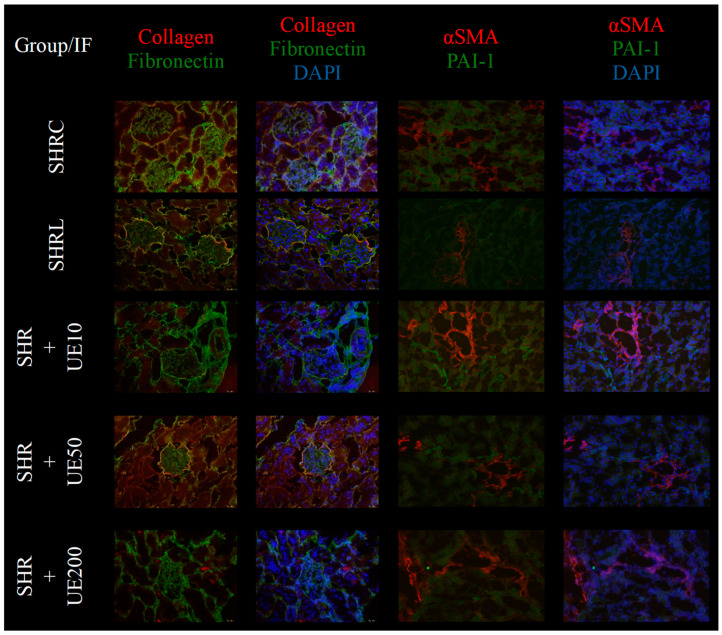
Collagen, fibronectin, α-SMA, and PAI-1 expression in kidneys of all investigated groups (magnification, ×200; α-SMA and PAI-1 of SHRL—magnification, ×100). The SHRC group exhibited slight collagen expressions within the kidney interstitium and mild expression within glomeruli, mostly localized in Bowman’s capsule. The expression of fibronectin was widespread in the interstitial compartment and within the glomeruli. The SHRL group exhibited decreased collagen and fibronectin expression within the kidney interstitium and within glomeruli. SHR+UE10 dose did not significantly reduce fibronectin expression, but collagen was prominently decreased. SHR+UE 50 dose significantly reduced fibronectin and collagen expression in the kidney interstitium, but with slightly preserved collagen expression in Bowman’s capsule. SHR+UE 200 dose completely reduced collagen expression in kidney structures, but without alteration in the expression of fibronectin compared to control and UE10 groups. SHRC and all investigated SHR+UE groups showed slight focal α-SMA expression within the interstitium, along with the normal expression in blood vessels, and also had focal interstitial PAI-1 staining. The SHRL group showed normal α-SMA expression localized to the area of blood vessels, with a reduced PAI-1 immunofluorescent staining. The SHR+UE10 group did not influence the α-SMA expression and PAI-1 expression, while SHR+UE50 and SHR+UE200 reduced expression of both molecules resembling the SHRL group. Nuclei were identified by 4,6-diamino-2-phenylindolyl-dihydrochloride (DAPI). SHRC—the control group, SHRL group—the SHR received losartan 10 mg/kg/day for 4 weeks; SHR+UE10, SHR+UE50, and SHR+UE200 groups—the SHR received 10, 50, and 200 mg/kg/day of UE for 4 weeks.

Among the investigated animals within each experimental group, the pattern of immunostaining was consistent, with no significant differences observed. The expression pattern was similar across all subjects within the group, demonstrating uniformity in the immunostaining results. In the control group (SHRC), collagen expression was mildly detected within the kidney interstitium and glomeruli, with predominant localization in Bowman’s capsule. Fibronectin expression was widespread, being present in both the interstitial compartment and glomeruli, indicating baseline matrix deposition. In the losartan-treated group (SHRL), both collagen and fibronectin expression were significantly reduced in the interstitium and glomeruli, demonstrating an antifibrotic effect of the treatment. The SHR+UE10 group did not significantly affect fibronectin expression, which remained comparable to the control group, but exhibited a prominent decrease in collagen expression, particularly in the interstitial compartment. The SHR+UE50 group, however, markedly reduced both collagen and fibronectin expression within the kidney interstitium. Despite this, a slight preservation of collagen expression was noted in Bowman’s capsule. The SHR+UE200 group showed a complete reduction in collagen expression across all kidney structures. However, this group did not show any significant changes in fibronectin expression compared to the SHRC and SHR+UE10 groups. Regarding α-SMA and PAI-1 expression, the SHRC group and all SHR+UE groups exhibited slight focal α-SMA expression within the interstitial regions, along with normal expression in blood vessels. Similarly, focal interstitial PAI-1 staining was noted. In the SHRL group, α-SMA expression remained normal and confined to vascular structures, while PAI-1 staining was markedly reduced, reflecting its anti-inflammatory effects. The SHR+UE10 group did not alter α-SMA or PAI-1 expression, maintaining levels comparable to the control. In contrast, the SHR+UE50 and SHR+UE200 groups showed reductions in both α-SMA and PAI-1 expressions, mirroring the pattern observed in the SHRL group (Figure 5).

## 3. Discussion

Hypertension is the main cause of kidney damage. In addition to the antihypertensive effect of UE, this study demonstrated for the first time the beneficial effects of UE on kidney function and structure in terms of reduced microalbuminuria, improved antioxidant capacity and oxidative stress in the adult SHR kidney. Our findings highlight a dose-dependent stimulation of eNOS, suppression of nNOS, and modulation of profibrotic and proinflammatory markers in response to UE treatments. We also showed that the Nrf-2 signaling mechanism and its downstream mediated antioxidant proteins, such as SOD and CAT, were not involved in the prevention of oxidative stress in the kidneys of UE-treated SHR. Chronic treatment with AT-1 receptor blocker (ARB) losartan reduces blood pressure, plasma NO_2_^−^, and kidney expression of nNOS, Nrf-2, SOD, CAT, collagen IV, FN, and PAI-1. Losartan increased the abundance of eNOS and 6-NO_2_Trp in the kidney of SHR, despite unchanged iNOS and improved kidney oxidative stress.

Previous studies from our group [19] and others [15,16,25] have shown that therapeutic benefits of UE could be attributed to its phenolic compounds. Here, the reduction in MAP in response to UE treatment follows the increase in the content of total nitrates and nitrites in plasma and urine. Until recently, food-derived nitrates and nitrites were considered as inert products of the food with potentially harmful effects on human health [26]. However, other studies have shown that these ions are physiologically “recycled” in the blood and tissues thus represent an exogenous source of NO [26,27], due to mechanisms involving the reduction in nitrates to nitrites by microbiota from saliva [26], as well as intestinal microflora [28], and conversion of nitrites into nitric acid in the stomach [26]. The observed high nitrite content of UE in the present study (14.72 mg/g), in addition to the abovementioned findings, could explain the increased concentration of NO metabolites in the plasma and urine of SHRs treated with UE.

In SHR+UE50 and SHR+UE200, we found dose-dependent increase in the expression of the eNOS enzyme, followed by a decrease in the expression of the nNOS enzyme in all treated groups, while the expression of the iNOS enzyme remained unchanged. This is not surprising considering that numerous in vitro and in vivo studies have shown that various plant extracts and products, as well as individual phenolic compounds, can modulate the expression, synthesis, and activity of enzymes from the NOS family [29,30,31,32]. The expression of nNOS enzyme in SHR rats is significantly increased compared to its content in the Wistar Kyoto (WKY) strain [33], and its overexpression is mediated by angiotensin II, i.e., by activating signaling pathways related to the AT1 receptor [34], which the authors confirmed using losartan at the same dose as we did in the present study (10 mg/kg BW). On the other hand, the reduction in kidney nNOS enzyme expression in SHR rats treated with all three UE dosages could be attributed to the strong antioxidant effects of the extract, because oxidative stress can also lead to an increase in the expression of this enzyme in the kidney [34].

ROS generated by oxidative stress is increased in hypertensive animals and humans [3,4,7], and the plasma antioxidant capacity, a measure of non-enzymatic antioxidant activity [19], is mostly exogenous in nature [35]. The increase in kidney antioxidant capacity of SHR+UE50 and SHR+UE200 could also be the reason for the reduction in lipid peroxidation through the direct neutralization of ROS including superoxide radical, hydroxyl radical, and hydrogen peroxide [19], primarily through the activity of phenolic compounds metabolites [36] present in our UE, namely, chlorogenic acid, 2-O-caffeoyl malic acid, and rutin [20]. This increased antioxidant capacity and reduced oxidative stress in those rats as well as in the SHRL group occurs despite the elevated 6-NO_2_Trp expression that has been observed. To the best of our knowledge, there is no literature data about losartan, UE-induced nitration of the protein containing tryptophan, or tryptophan alone. The modification of tryptophan residues in proteins may occur at a more limited number of sites in vivo than that of tyrosine residues and may result in modulation of the specific interaction of proteins and enzymes with other molecules [5]. Some actions of radicals and oxidants such as oxidation are reversible, while others, such as nitration or breaking of the histidine and tryptophan rings, are irreversible, resulting in altered conformation and modified turnover of targeted protein [37]. Skorstengaard and collages showed that FN, a multiple-domain glycoprotein secreted by many cell types, is composed of two identical/nearly identical polypeptide chains, linked by two disulfide bridges near the C terminus containing tryptophan amino acid in this amino-terminal domain [38], and that polymerization of secreted FN precedes the deposition and maturation of other ECM proteins (including collagen) [39]. In addition, PAI-1, a member of the serine protease superfamily inhibitor (serpin) with antiprotease activity, plays a pivotal role in various acute and chronic pathophysiological processes, including cardiovascular disease, tissue fibrosis, cancer, and age-related diseases [40]. We did not find any changes in PAI-1 expression in the UE10-treated group, while treatments with UE 50 and 200 mg/kg/day reduced both α-SMA and PAI-1 expressions. Verheyden et al. illustrated the importance of tryptophan residues for the kinetics of the conformational change in PAI-1, and that tryptophan residues 262 and 175 influence the transition from the active to the latent conformation [41]. Considering the abovementioned, we suggested that the PAI-1 expression observed in our study after UE treatment represents a latent rather than active form. If such post-translational modifications of tryptophan residues of FN and PAI-1 occur in our study, we assume that they could affect various cellular processes, thus resulting in regression of fibrogenesis and inflammation in SHR. Furthermore, renal function assessed by microalbuminuria in all UE groups was improved. Furthermore, the UE-induced decrease in PAI-1 expression was similar to that in losartan-treated SHRs, in which we noted a decrease in collagen IV, FN, and SMA, indicating an antifibrotic effect of the treatment. Porteri et al. [42] previously demonstrated that renal collagen type IV content is increased in SHR glomeruli compared to WKY and becomes normalized after ACE or ARB treatment (even at low, non-hypotensive doses) suggesting a pressure-independent protective effect of these drugs.

It is well known that in response to oxidative stress, Nrf2 is activated and binds to antioxidant response elements to activate the transcription and translation of antioxidant genes and proteins, respectively, while the inhibition of Nrf2 expression affects the expression level of antioxidant proteins [43], among them SOD and CAT. Erejuwa et al. found markedly downregulated Nrf2 mRNA expression level in the kidney of SHR compared with WKY, followed by the increased malondialdehyde content, despite the elevated CAT activity [44]. Results that we reported here show an equal reduction in Nrf-2/SOD/CAT molecule expressions in the kidney of SHR exposed to chronic losartan and UE50 treatment. However, both treatments successfully improved oxidative stress. Such discrepancy indicates a significant role of mechanisms other than Nrf2 signaling, for instance, blockade of AT1 stimulated production of ROS by NADPH oxidase [45], or direct antioxidant activity of UE [19] in reducing ROS production under these experimental conditions.

## 4. Materials and Methods

### 4.1. Plant Material

Plant material was acquired from the Institute for Medicinal Plant Research “Dr. Josif Pančić”, Belgrade, Serbia. It was harvested at the mountain Jastrebac, Serbia (batch number 20040716, quality control number 1629).

### 4.2. Preparation of Extract

The UE was prepared as previously described [19]. Briefly, dried and milled *Urtica dioica* L. leaves were extracted with ultrasound-assisted extraction technique under previously determined optimal conditions for maximal total phenolic content (54% *v*/*v* aqueous-methanol as solvent, 38 min extraction time, and 1:20 solid to liquid ratio). The extract was filtered, and methanol evaporated under a vacuum (IKA^®^ Werke GmbH & Co., Staufen im Breisgau, Germany). The extract was pre-frozen in deep freeze at −80 °C for 1 h and then lyophilized. Conditions of the lyophilization were −60 °C, 0.011 bar, for 24 h, and −75 °C, 0.0012 bar, for 4 h (Beta 1–8 Freeze Dryer, Martin Christ, Osterode am Harz, Germany). Part of the extract was used for the separation, detection, and quantification of phenolic compounds present in this extract where the content of chlorogenic acid, 2-O-caffeoyl malic acid, and rutin in UE was determined to be 15.3, 12.3, and 6 mg/g UE, respectively, by HPLC [19]. The remaining dry extract (UE) was used for the present in vivo study.

### 4.3. Ethical Statement

The experimental protocol was approved by the Ethics Committee of the Institute for Medical Research, University of Belgrade, as well as by the Veterinary Directorate, Ministry of Agriculture and Environmental Protection, Republic of Serbia (323-07-02449/2014-05) according to the National Law on Animal Welfare (‘Službeni Glasnik’ No. 41/09, 39/10), which is consistent with guidelines for animal research and principles of the European Convention for the Protection of Vertebrate Animals Used for Experimental and Other Purposes (Official Daily N. L 358/1-358/6, 18 December 1986), and the directive on the protection of animals used for scientific purposes (Directive 2010/63/EU of the European Parliament and of the Council, 22 September 2010).

### 4.4. Animal Studies

In this study, fifty male spontaneously hypertensive rats (SHR), six months old and weighing approximately 300 g, were used. The animals were bred at the Institute for Medical Research, University of Belgrade, Serbia, and were fed with standard chow for laboratory rats (AGRO-FIRM d.o.o., Požarevac, Serbia) with food and water at disposal ad libitum until the end of the experiment. Animals were kept in groups of four rats per cage with 12 h light/dark cycles at room temperature. Animals were divided into 5 experimental groups which in the course of four weeks received orally 0.5 mL/day of water (control group SHRC), conventional antihypertensive drug—losartan (DUP 153, Du Pont, Wilmington, DE, USA) 10 mg/kg/day in the volume of 0.5 mL of water (SHRL), and 10, 50, and 200 mg/kg/day of UE (SHR+UE10, SHR+UE50, and SHR+UE200, respectively) dissolved in 0.5 mL of water.

### 4.5. Body Weight, Urine Flow, and Blood Pressure Measurement

At the end of the treatment period, the body weight of all rats was measured, and rats were placed in metabolic cages for 24-h urine collection. Urine flow (Uf, mL/24 h) was calculated from urine volume normalized to body weight (BW). Next, rats were anesthetized with 35 mg/kg BW sodium pentobarbital intraperitoneally. For mean arterial blood pressure (MAP) measurements, the left femoral artery was cannulated with PE-50 catheters (Clay-Adams, Parsippany, NY, USA) and connected to the physiological data acquisition system (9800TCR Cardiomax III-TCR, Columbus, OH, USA).

### 4.6. Sample Collection and Preparation

Blood samples were collected using lithium-heparin (Sigma-Aldrich, St. Louis, MO, USA) as an anticoagulant. Blood was centrifuged at 4000 revolutions per minute (Heraeus Megafuge 1.0 R, Heraeus, Hanau, Germany) for 20 min, and plasma was kept at −20 °C until further analysis. Kidneys were removed, washed in ice-cold saline (0.9% *m*/*w* NaCl), and weighed (kidney weight, KW). KW/BW ratio was calculated and expressed as g/kg. One part of the longitudinally dissected kidney was stored at −80 °C until assaying.

### 4.7. Assessment of Kidney Function

Plasma creatinine (pCr) and urine albumin concentrations (uAlb) were measured by an automatic COBAS INTEGRA 400 plus analyzer (Hoffmann-La Roche, Leitch Diagnostic, Penzberg, Germany). Urinary albumin excretion (Albexc) was calculated from the Uf and urine albumin concentration and expressed as mg/24 h.

### 4.8. Determination of Plasma, Urine, and Kidney NO Metabolites

Total nitrate/nitrite (NO_x_) and nitrite (NO_2_^−^) concentrations were measured in plasma, urine, and kidney homogenate by the Griess reagent method [46]. Briefly, diluted samples were deproteinized by adding 5 μL 15 g/L of ZnSO_4_. For the determination of NO_x_, 50 μL of each sample was added to a microplate with 30 μL 8 μM of flavin adenine dinucleotide (FAD), 10 μL 1 mM of β-Nicotinamide adenine dinucleotide phosphate (NADPH), and 10 μL of nitrate reductase (10 U/mL). After the reduction in nitrate to nitrite, 100 μL of Griess reagent (120 mM of sulfanilamide, 2.5% phosphoric acid, and 8 mM of N-1-naphthylethylenediamine) was added to these samples, as well as to non-reductase-treated samples. The absorbance of all samples was measured at 540 nm after 10 min of incubation in the dark and at room temperature, and results are expressed as micromoles per liter (μmol/L) for plasma and urine and micromole per milligram of kidney tissue (μmol/mg).

### 4.9. Determination of NO_2_^−^ Content in UE

Griess reagent was also used for the determination of NO_2_^−^ content in UE, and the result was expressed as milligrams per gram of UE (mg/g).

### 4.10. Lipid Peroxidation

Thiobarbituric acid-reactive substance (TBARS) assay was used for the estimation of lipid peroxidation in the kidney (kTBARS) [47]. It was achieved by mixing 0.4 mL of the sample with 0.2 mL of 28% trichloroacetic acid and centrifuging the mixture for 4 min at 15,000 RPM. The supernatant was collected and mixed with 0.1 mL 694 mM of thiobarbituric acid and incubated at 100 °C for 15 min. Readings were performed at 540 nm. Results are expressed as nanomoles per milligram of kidney tissue (nmol/mg).

### 4.11. Advanced Oxidation Protein Products

Advanced oxidation protein products in the kidney (kAOPP) were measured according to the previously described method [48]. In brief, two hundred microliters of sample were placed in a well of a 96-well microtiter plate, followed by 20 µL of acetic acid. Then, 10 µL of 1.16 M KI was added, as well as 20 µL of acetic acid. The absorbance was measured at 340 nm. Results are expressed as micromoles of chloramine-T equivalents per gram of kidney tissue (µmol/g).

### 4.12. Antioxidant Status

Antioxidant status in the kidney (kABTS) was measured using the Trolox equivalent antioxidant capacity method [49]. Briefly, 5 mL of 7 mM ABTS solution was mixed with 0.1 mL of 125 mM K2S2O8 solution and left in the dark for 12–16 h. The absorbance of the ABTS•+ solution was adjusted to 0.70 at 734 nm with 50 mM PBS (pH 7.4). After the addition of 2.0 mL of diluted ABTS•+ reagent to 20 μL of kidney homogenate, the reaction mixture was incubated for 6 min at 30 °C and the absorbance was recorded at 734 nm. Results are expressed as micromoles of Trolox equivalents per gram of kidney tissue (µmol TE/g).

### 4.13. Western Blot Analysis

Kidney tissue samples (6 animals per group) were homogenized in RIPA buffer, as previously described [50]. Briefly, cold RIPA lysis buffer (50 mM Tris-HCl pH 7.5, 150 mM NaCl, 1% Triton x-100, 1% sodium deoxycholate, 0.1% sodium dodecyl sulfate, 2 mM EDTA, and 50 mM NaF) with an added protease inhibitor cocktail (Thermo Scientific, Pierce Biotechnology, Rockford, IL, USA) and sodium orthovanadate (sample:buffer = 1:10 *m*/*w*), was used for kidney homogenization. Lysates of the kidneys were incubated at 4 °C for 20 min, and then centrifuged for 20 min at 15,000× *g*, 4 °C. Protein concentration in samples was determined by the BCA Protein Assay Kit (Thermo Scientific, Pierce Biotechnology, Rockford, lL, USA). Equal amounts of protein were separated by SDS-PAGE and transferred to nitrocellulose membranes (Appli-Chem GmbH, Darmstadt, Germany). Membranes were then probed with primary antibodies: eNOS (1:1000, SAB4502016, Sigma-Aldrich, Merck KGaA, Darmstadt, Germany), iNOS (1:1000, SAB4502011, Sigma-Aldrich, Merck KGaA, Darmstadt, Germany), nNOS (1:1000, AB5380, Millipore, Merck KGaA, Darmstadt, Germany), and actin (1:1000, A5060, Sigma-Aldrich, Merck KGaA, Darmstadt, Germany). Peroxidase-conjugated goat anti-rabbit immunoglobulin (1:40000, A0545, Sigma-Aldrich, Merck KGaA, Darmstadt, Germany) was used as a secondary antibody. The eNOS, iNOS, and nNOS protein bands were visualized using an enhanced chemiluminescence reagent system (GE Healthcare, Amersham, UK) and quantified by the Image Master Total Lab TL120 (GE Healthcare) densitometry software TotalLab Control Centre v2009. Next, a set of membranes was tested with primary antibodies against 6-nitrotryptophan (6-NO_2_Trp, 1:1000, ab243072, Abcam, Cambridge, UK), Nrf-2 (1:500, Novus Biologicals NBP1-32822, Centennial, CO, USA), SOD (1:1000, ab16831, Abcam, Cambridge, UK), CAT (1:1000, ab16731, Abcam, Cambridge, UK), and normalized to actin (1:1000, A5060, Sigma-Aldrich, Merck KGaA, Darmstadt, Germany). Incubation with horseradish peroxidase-conjugated secondary antibodies followed (anti-rabbit IgG, 1:40,000, A0545, Sigma-Aldrich, Merck KGaA, Darmstadt, Germany for SOD, CAT, and Nrf-2; anti-mouse IgG, 1:2500, A5278, Sigma-Aldrich, Merck KGaA, Darmstadt, Germany for 6-NO_2_Trp), after which a chemiluminescence reagent was applied. Bands were visualized using the ChemiDoc Imaging system (Bio-Rad Laboratories, Inc., Hercules, CA, USA), and analyzed in Image Lab v6.0.1. software. At least three independent immunoblot experiments were carried out for each examined protein expression.

### 4.14. Immunofluorescent Staining

Immediately after removal, kidney tissue was snap-frozen in liquid nitrogen for consequent use for routine immunofluorescent analysis, and the rest of the tissue was stored at −80 °C for further immunostaining. Five μm thick cryostat sections were dried for 1h at room temperature, and fixed in acetone for 10 min. To obtain double fluorescent labeling of collagen IV/fibronectin and SMA/PAI-1, we applied the following primary antibodies: collagen IV clone CIV22 (mouse monoclonal, 1:50, Bio SB, Goleta, CA, USA), fibronectin ab2413 (rabbit polyclonal, 1:100, Abcam), SMA clone 1 A4 (mouse monoclonal, 1:400, DAKO), and PAI-1 cat no NBP119773 (rabbit polyclonal, 1:20 Novus Biologicals, Centennial, CO, USA). Secondary antibodies were the following goat anti-rabbit IgG (H+L) Alexa Flor 488 (1:500, Life Technologies, Thermo Fisher Scientific Inc., Waltham, MA, USA) and goat anti-mouse IgG (H+L) Alexa Flor 647 (1:100, Abcam, Cambridge, UK). Nuclei were identified by 4,6-diamino-2-phenylindolyl-dihydrochloride (DAPI; 1 μg/mL). Sections were mounted with Fluoro Preserve Reagent (Calboichem, Darmstadt, Germany). Slides were analyzed on fluorescence microscopy (Olympus AX70, Olympus Optical Co., Ltd., Tokyo, Japan) with the digital camera Olympus DP74 (20.7 Mpx, 1/1.2 inch) and computer-supported imaging system cellSens Standard (CS-ST-V2). During the analysis of the microscope slides, the pathologist was blinded to the experimental protocol, meaning he was unaware of which experimental group the slides belonged to. The only information available was the specific antibodies being analyzed and the fluorescent color marking them. For each individual case, the pathologist recorded the pathological compartments where expression was observed, noting whether it was diffuse or focal, and indicating the intensity of the expression.

### 4.15. Statistical Analysis

The results are presented as the mean values with standard error of the mean (SEM). All measurements were performed in triplicate. For comparison between different groups, we used a one-way ANOVA omnibus test with Fisher least significant difference (LSD) post hoc test (Statistica version 8.0., StatSoft Inc., Tulsa, OK, USA). A *p*-value of less than 0.05 was considered statistically significant.

## 5. Conclusions

In this study, UE in a dose of 10 mg/kg/day failed to improve hypertension, oxidative stress, and ECM remodeling in SHR, although albuminuria became reduced and tryptophan nitration was increased. The dose UE50 significantly reduced MAP, albuminuria, oxidative stress, fibroinflammatory markers, and NRF-2/CAT/SOD signaling, with a significant increase in 6-nitrotryptophan and eNOS expressions compared to the control. The observed effects of UE were dose-dependent in part; however, UE200 did not exceed the net benefit achieved after 4-week UE50 administration. The beneficial effects of losartan, as well as that of UE, were independent of NRF-2 signalization in SHR. Interestingly, all treatments induced the increase in 6-nitrotryptophan expression, indicating the necessity of further studies to elucidate the modulatory mechanisms of such nitrated tryptophan.

## Figures and Tables

**Table 1:** Body weight (BW), urine flow (Uf), kidney weight (KW), kidney weight to body weight ratio (KW/BW), plasma creatinine concentration (pCr), albumin excretion (Albexc), and mean arterial pressure in SHR. SHRC received 0.5 mL/day of water, SHR+L received 10 mg/kg/day of losartan, SHR+UE10, SHR+UE50, and SHR+UE200 received 10, 50, and 200 mg/kg/day of *Urtica dioica* L. leaf extract (UE), respectively.

	SHRC	SHRL	SHR+UE10	SHR+UE50	SHR+UE200
BW, g	304 ± 5	301 ± 6	321 ± 6 ^#^	304 ± 5	312 ± 4
Uf, mL/24 h	10.0 ± 1.1	12.2 ± 0.9	7.5 ± 0.6 ^##^	10.7 ± 1.1	8.6 ± 0.6
KW, g	1.05 ± 0.03	1.06 ± 0.03	1.07 ± 0.02	1.06 ± 0.01	1.09 ± 0.03
KW/BW	0.348 ± 0.006	0.351 ± 0.007 ^#^	0.329 ± 0.006	0.349 ± 0.005	0.350 ± 0.009
pCr, µmol/mL	43.7 ± 1.7	49.6 ± 1.8 **	46.3 ± 1.6	42.9 ± 1.3	45.5 ± 1.2
Alb_exc_, mg/24 h	0.422 ± 0.046	0.450 ± 0.041	0.194 ± 0.030 ***^,###^	0.317 ± 0.033 *^,#^	0.267 ± 0.024 **^,##^
MAP, mmHg	162 ± 4	135 ± 5 **	150 ± 9	143 ± 8 *	141 ± 8 *

* *p* < 0.05, ** *p* < 0.01, and *** *p* < 0.001 compared to SHRC, # *p* < 0.05, ## *p* < 0.01, and ### *p* < 0.001 compared to SHRL. Data presented as mean ± SEM (one-way ANOVA omnibus test with Fisher least significant difference (LSD) post hoc test, Statistica version 8.0., StatSoft Inc., Tulsa, OK, USA).

## Data Availability

The data presented in this study are available within the article.

## References

[1] Kodavanti U.P., Schladweiler M.C., Ledbetter A.D., Watkinson W.P., Campen M.J., Winsett D.W., Richards J.R., Crissman K.M., Hatch G.E., Costa D.L. (2000). The spontaneously hypertensive rat as a model of human cardiovascular disease: Evidence of exacerbated cardiopulmonary injury and oxidative stress from inhaled emission particulate matter. Toxicol. Appl. Pharmacol..

[2] Mihailović-Stanojević N., Varagić J., Jovović D., Miloradović Z., Marković-Lipkovski J., Jerkić M. (2003). Aging and hypertension as factors of progressive renal failure. Med. Pregl..

[3] Chien S.-J., Lin K.-M., Kuo H.-C., Huang C.-F., Lin Y.-J., Huang L.-T., Tain Y.-L. (2014). Two different approaches to restore renal nitric oxide and prevent hypertension in young spontaneously hypertensive rats: L-citrulline and nitrate. Transl. Res..

[4] Hermann M., Flammer A., Lüscher T.F. (2006). Nitric Oxide in Hypertension. Clin. Hypertens..

[5] Yamakura F., Ikeda K. (2006). Modification of tryptophan and tryptophan residues in proteins by reactive nitrogen species. Nitric Oxide.

[6] Wei W., Ma N., Fan X., Yu Q., Ci X. (2020). The Role of Nrf2 in Acute Kidney Injury: Novel Molecular Mechanisms and Therapeutic Approaches. Free. Radic. Biol. Med..

[7] Tanase D.M., Apostol A.G., Costea C.F., Tarniceriu C.C., Tudorancea I., Maranduca M.A., Floria M., Serban I.L. (2022). Oxidative Stress in Arterial Hypertension (HTN): The Nuclear Factor Erythroid Factor 2-Related Factor 2 (Nrf2) Pathway, Implications and Future Perspectives. Pharmaceutics.

[8] Donnelly R., Collinson D.J., Manning G. (2003). Hypertension, matrix metalloproteinases and target organ damage. J. Hypertens..

[9] Lekgabe E.D., Kiriazis H., Zhao C., Xu Q., Moore X.L., Su Y., Bathgate R.A., Du X.-J., Samuel C.S. (2005). Relaxin reverses cardiac and renal fibrosis in spontaneously hypertensive rats. Hypertension.

[10] Genovese F., Manresa A.A., Leeming D.J., Karsdal M.A., Boor P. (2014). The extracellular matrix in the kidney: A source of novel non-invasive biomarkers of kidney fibrosis?. Fibrogenesis Tissue Repair.

[11] Mihailovic-Stanojevic N., Jovovic D., Miloradovic Z., Drndarevic N., Markovic-Lipkovski J., Jerkic M., Heinle H., Schulteand H., Hahmann H. (2004). Long-Term Losartan But Not Bosentan Treatment Revert Vascular Injury in The Kidney of Aged Spontaneously Hypertensive Rats. Besondere Aspekte der Cerebrovaskulären und Peripheren Arteriosklerose—17. Jahrestagung der Deutschen Gesellschaft für Arterioskleroseforschung 2003.

[12] Ma L.-J., Fogo A.B. (2009). PAI-1 and kidney fibrosis. Front. Biosci (Landmark Ed)..

[13] Abrahamsen C.T., Pullen M.A., Schnackenberg C.G., Grygielko E.T., Edwards R.M., Laping N.J., Brooks D.P. (2002). Effects of Angiotensins II and IV on Blood Pressure, Renal Function, and PAI-1 Expression in the Heart and Kidney of the Rat. Pharmacology.

[14] Legssyer A., Ziyyat A., Mekhfi H., Bnouham M., Tahri A., Serhrouchni M., Hoerter J., Fischmeister R. (2002). Cardiovascular effects of *Urtica dioica* L. in isolated rat heart and aorta. Phytother. Res..

[15] Qayyum R., Qamar H.M.U.D., Khan S., Salma U., Khan T., Shah A.J. (2016). Mechanisms underlying the antihypertensive properties of *Urtica dioica*. J. Transl. Med..

[16] Dizaye K.F., Alberzingi B.O., Sulaiman S.R. (2013). Renal and Vascular Studies of Aqueous Extract of *Urtica dioica* in Rats and Rabbits. Iraqi J. Vet. Sci..

[17] Tahri A., Yamani S., Legssyer A., Aziz M., Mekhfi H., Bnouham M., Ziyyat A. (2000). Acute Diuretic, Natriuretic and Hypotensive Effects of a Continuous Perfusion of Aqueous Extract of *Urtica dioica* in the Rat. J. Ethnopharmacol..

[18] Testai L., Chericoni S., Calderone V., Nencioni G., Nieri P., Morelli I., Martinotti E. (2002). Cardiovascular Effects of *Urtica dioica* L. (Urticaceae) Roots Extracts: In vitro and in vivo Pharmacological Studies. J. Ethnopharmacol..

[19] Vajic U.-J., Grujic-Milanovic J., Miloradovic Z., Jovovic D., Ivanov M., Karanovic D., Savikin K., Bugarski B., Mihailovic-Stanojevic N. (2018). *Urtica dioica* L. leaf extract modulates blood pressure and oxidative stress in spontaneously hypertensive rats. Phytomedicine.

[20] Vajić U.-J., Grujić-Milanović J., Živković J., Šavikin K., Gođevac D., Miloradović Z., Bugarski B., Mihailović-Stanojević N. (2015). Optimization of extraction of stinging nettle leaf phenolic compounds using response surface methodology. Ind. Crops Prod..

[21] Vajic U.-J., Zivkovic J., Ivanov M., Jovovic D., Savikin K., Bugarski B., Mihailovic-Stanojevic N. (2022). Optimization of the extraction of antioxidants from stinging nettle leaf using response surface methodology. Maced. J. Chem. Chem. Eng..

[22] Salih N.A. (2015). Effect of nettle (*Urtica dioica*) extract on gentamicin induced nephrotoxicity in male rabbits. Asian Pac. J. Trop. Biomed..

[23] Hilal H., El-Kholie E., Mousa S. (2024). Protective Effect of Nettle (*Urtica dioica*), Leaves and Seeds on Kidney Disorder in Gentamicin-Induced Rats. J. Home Econ.-Menofia Univ..

[24] Zhang Z. (2024). Hypertensive Arteriolar Nephrosclerosis. https://www.msdmanuals.com/home/kidney-and-urinary-tract-disorders/blood-vessel-disorders-of-the-kidneys/hypertensive-arteriolar-nephrosclerosis.

[25] Ziyyat A., Legssyer A., Mekhfi H., Dassouli A., Serhrouchni M., Benjelloun W. (1997). Phytotherapy of hypertension and diabetes in oriental Morocco. J. Ethnopharmacol..

[26] Lundberg J.O., Weitzberg E., Gladwin M.T. (2008). The nitrate–nitrite–nitric oxide pathway in physiology and therapeutics. Nat. Rev. Drug Discov..

[27] Hord N.G., Tang Y., Bryan N.S. (2009). Food sources of nitrates and nitrites: The physiologic contact for potential health benefits. Am J Clin Nutr.

[28] Jensen F.B. (2009). The role of nitrite in nitric oxide homeostasis: A comparative perspective. Biochim. Biophys. Acta Bioenerg..

[29] Ugusman A., Zakaria Z., Chua K.H., Nordin N.A.M.M., Mahdy Z.A. (2014). Role of rutin on nitric oxide synthesis in human umbilical vein endothelial cells. Sci. World J..

[30] Wallerath T., Poleo D., Li H., Förstermann U. (2003). Red wine increases the expression of human endothelial nitric oxide synthase: A mechanism that may contribute to its beneficial cardiovascular effects. J. Am. Coll. Cardiol..

[31] Abd-Elbaset M., Arafa E.-S.A., El Sherbiny G.A., Abdel-Bakky M.S., Elgendy A.N.A.M. (2015). Quercetin modulates iNOS, eNOS and NOSTRIN expressions and attenuates oxidative stress in warm hepatic ischemia-reperfusion injury in rats. Beni-Suef Univ. J. Basic. Appl. Sci..

[32] Leikert J.F., Räthel T.R., Wohlfart P., Cheynier V., Vollmar A.M., Dirsch V.M. (2002). Red wine polyphenols enhance endothelial nitric oxide synthase expression and subsequent nitric oxide release from endothelial cells. Circulation.

[33] Tojo A., Onozato M.L., Fujita T. (2006). Role of macula densa neuronal nitric oxide synthase in renal diseases. Med. Mol. Morphol..

[34] Pereira T.M.C., Balarini C.M., Silva I.V., Cabral A.M., Vasquez E.C., Meyrelles S.S. (2009). Endogenous angiotensin II modulates nNOS expression in renovascular hypertension. Braz. J. Med. Biol. Res..

[35] Kampa M., Nistikaki A., Tsaousis V., Maliaraki N., Notas G., Castanas E. (2002). A new automated method for the determination of the Total Antioxidant Capacity (TAC) of human plasma, based on the crocin bleaching assay. BMC Clin. Pathol..

[36] Devaraj S., Vega-lópez S., Kaul N., Schönlau F., Rohdewald P., Jialal I. (2002). Supplementation with a pine bark extract rich in polyphenols increases plasma antioxidant capacity and alters the plasma lipoprotein profile. Lipids.

[37] Ruiz-Ojeda F.J., Olza J., Gil Á., Aguilera C.M. (2018). Oxidative Stress and Inflammation in Obesity and Metabolic Syndrome. Obesity: Oxidative Stress and Dietary Antioxidants.

[38] Skorstengaard K., Thøgersen H.C., Petersen T.E. (1984). Complete primary structure of the collagen-binding domain of bovine fibronectin. Eur. J. Biochem..

[39] Bowers S.L.K., Davis-Rodriguez S., Thomas Z.M., Rudomanova V., Bacon W.C., Beiersdorfer A., Ma Q., Devarajan P., Blaxall B.C. (2019). Inhibition of fibronectin polymerization alleviates kidney injury due to ischemia-reperfusion. Am. J. Physiol. Renal Physiol..

[40] Sillen M., Declerck P.J. (2020). Targeting PAI-1 in Cardiovascular Disease: Structural Insights Into PAI-1 Functionality and Inhibition. Front. Cardiovasc. Med..

[41] Verheyden S., Sillen A., Gils A., Declerck P.J., Engelborghs Y. (2003). Tryptophan properties in fluorescence and functional stability of plasminogen activator inhibitor. Biophys. J..

[42] Porteri E., Rodella L., Rizzoni D., Rezzani R., Paiardi S., Sleiman I., De Ciuceis C., Boari G.E.M., Castellano M., Bianchi R. (2005). Effects of olmesartan and enalapril at low or high doses on cardiac, renal and vascular interstitial matrix in spontaneously hypertensive rats. Blood Press..

[43] Jin Q., Zhu Q., Wang K., Chen M., Li X. (2021). Allisartan isoproxil attenuates oxidative stress and inflammation through the SIRT1/Nrf2/NF-κB signalling pathway in diabetic cardiomyopathy rats. Mol. Med. Rep..

[44] Erejuwa O., Sulaiman S., Suhaimi M., Sirajudeen K., Salleh S., Gurtu S. (2011). Impaired Nrf2-ARE pathway contributes to increased oxidative damage in kidney of spontaneously hypertensive rats: Effect of antioxidant (honey). Int. J. Cardiol..

[45] Vallés P.G., Bocanegra V., Costantino V.V., Lorenzo A.F.G., Benardon M.E., Cacciamani V. (2020). The renal antioxidative effect of losartan involves heat shock protein 70 in proximal tubule cells. Cell Stress Chaperones.

[46] Green L.C., Wagner D.A., Glogowski J., Skipper P.L., Wishnok J.S., Tannenbaum S.R. (1982). Analysis of nitrate, nitrite, and [^15^N]nitrate in biological fluids. Anal. Biochem..

[47] Ohkawa H., Ohishi N., Yagi K. (1979). Assay for lipid peroxides in animal tissues by thiobarbituric acid reaction. Anal. Biochem..

[48] Selmeci L., Seres L., Antal M., Lukács J., Regöly-Mérei A., Acsády G. (2005). Advanced oxidation protein products (AOPP) for monitoring oxidative stress in critically ill patients: A simple, fast and inexpensive automated technique. Clin. Chem. Lab. Med..

[49] Re R., Pellegrini N., Proteggente A., Pannala A., Yang M., Rice-Evans C. (1999). Antioxidant activity applying an improved ABTS radical cation decolorization assay. Free Radic. Biol. Med..

[50] Karanovic D., Grujic-Milanovic J., Miloradovic Z., Ivanov M., Jovovic D., Vajic U.-J., Zivotic M., Markovic-Lipkovski J., Mihailovic-Stanojevic N. (2016). Effects of single and combined losartan and tempol treatments on oxidative stress, kidney structure and function in spontaneously hypertensive rats with early course of proteinuric nephropathy. PLoS ONE.

